# Effect of Gamma Irradiation on Cannabinoid, Terpene, and Moisture Content of Cannabis Biomass

**DOI:** 10.3390/molecules28237710

**Published:** 2023-11-22

**Authors:** Chandrani G. Majumdar, Mahmoud A. ElSohly, Elsayed A. Ibrahim, Mostafa A. Elhendawy, Donald Stanford, Suman Chandra, Amira S. Wanas, Mohamed M. Radwan

**Affiliations:** 1 National Center for Natural Products Research, School of Pharmacy, University of Mississippi, University, MS 38677, USA; cgon@olemiss.edu (C.G.M.); melsohly@olemiss.edu (M.A.E.); eibrahim@olemiss.edu (E.A.I.); suman@olemiss.edu (S.C.); aswanas@olemiss.edu (A.S.W.); 2 Department of Pharmaceutics and Drug Delivery, School of Pharmacy, University of Mississippi, University, MS 38677, USA; 3 Department of Pharmaceutical Analytical Chemistry, Faculty of Pharmacy, Suez Canal University, Ismailia 41522, Egypt; 4 Department of Chemistry and Biochemistry, University of Mississippi, University, MS 38677, USA; maelhend@olemiss.edu; 5 Department of Agriculture Biotechnology, Faculty of Agriculture, Damietta University, Damietta 34511, Egypt

**Keywords:** cannabis, gamma irradiation, cannabinoids, terpenes, moisture content

## Abstract

In recent years, cannabis has been proposed and promoted not only as a medicine for the treatment of a variety of illnesses, but also as an industrial crop for different purposes. Being an agricultural product, cannabis inflorescences may be contaminated by environmental pathogens at high concentrations, which might cause health problems if not controlled. Therefore, limits have to be placed on the levels of aerobic bacteria as well as yeast and mold. To ensure the safety of cannabis plant material and related products, a remediation process has to be put in place. Gamma irradiation is a sterilization process mainly used for pharmaceuticals, foods, cosmetics, agricultural, and herbal products including cannabis plant material. This study was designed to determine the effect of irradiation on the microbial count as well as on the chemical and physical profiles of the cannabis biomass, particularly cannabinoids, terpenes, and moisture content. The full cannabinoid profile was measured by GC/FID and HPLC analysis, while terpene profile and moisture content were determined using GC/MS and Loss on Drying (LoD) methods, respectively. Analyses were conducted on the samples before and after gamma irradiation. The results showed that the minimum and maximum doses were 15 and 20.8 KiloGray (KGY), respectively. Total Aerobic Microbial Count (TAMC) and Total Yeast and Mold Count (TYMC) were determined. The study showed that irradiation has no effect on the cannabinoids and little effect on terpenes and moisture content, but it did result in the virtual sterilization of the plant material, as evidenced by the low levels of bacterial and fungal colony-forming units (CFUs) < 10 after gamma irradiation.

## 1. Introduction

Cannabis (*Cannabis sativa* L.) is an annual flowering plant indigenous to Central Asia and has a rich and a fascinating recorded history [1]. Cannabis has played a significant role in many cultures for recreational and medicinal purposes and as a source of industrial fibers [2].

The dried resinous flowering buds of the cannabis plant are commonly known as “marijuana” [3].

In recent years, research on hemp has been progressively growing due to its use in various products, such as composites, plastics, and lubricants. Hemp has been used to produce textiles and fiber, in building materials like hempcrete and as a source of biofuel. Furthermore, hemp seeds can serve as a rich source of nutrition in different food products [4,5,6,7,8,9].

Cannabis contains hundreds of secondary metabolites, mainly cannabinoids, in addition to phenolic compounds, mono- and sesquiterpenoids, flavonoids, and alkaloids [10]. The unique chemicals (cannabinoids) include different types and concentrations, particularly THC, which is the main psychotropic cannabinoid and is responsible for the plant’s mind-altering effects [11]. Other cannabinoids including cannabidiol (CBD), cannabigerol (CBG), cannabichromene (CBC), and other minor compounds have comparable therapeutic benefits to THC without its side effects, as these cannabinoids could be used in the treatment of glaucoma, chronic musculoskeletal pain, spasm, nausea, spasticity of multiple sclerosis, and many others [12,13,14]. Cannabis can be divided into three main chemotypes, namely THC-dominant (type I), THC/CBD intermediate (type II), and CBD-dominant (type III) [15].

As an agricultural product, cannabis can be exposed to contamination with pathogenic fungi, bacteria, yeast, and mold during growing, drying, packing, and/or delivery, which may put consumers at health risk [16,17]. Fatal pulmonary Aspergillosis has been reported in some immunocompromised patients treated with inhaled marijuana [18,19]. Also, multistate outbreak enteritis caused by *Salmonella muenchen* has been reported [20]. Furthermore, *Clostridium botulinum* has been identified in some cannabis extracts [21].

Since cannabis legalization has progressed dramatically in recent years, and several cannabis-based products are now flooding the US market, contaminated cannabis may be found among these products, rendering them unsafe for consumers [22].

Although the federal government in the United States still considers cannabis illegal [23], nineteen U.S. states have legalized marijuana for recreational use [24]. Furthermore, a total of 37 states, three territories, and the District of Columbia allow cannabis products to be used medically [25].

Recently, the United States Food and Drug Administration (FDA) published guidance on cannabis and cannabis-derived products in which it is recommended that these products should be regulated by the same standards as any other botanical raw material, botanical drug substance, or botanical drug product [26]. In addition, many states provide their own guidelines to protect cannabis consumers for either medicinal, recreational, or both purposes [27]. Consequently, cannabis and cannabis-based products have to be free from contaminants—pesticides, microbes, molds, bacteria, heavy metals, and solvents—to protect the safety of cannabis consumers.

Several approved technologies are commonly used to reduce microbial contamination in foods and pharmaceutical products, including ethylene oxide gas, gamma irradiation, and X-ray irradiation [28]. Gamma irradiation remains the recommended method for decontaminating herbal products including cannabis [29]. However, in addition to gamma irradiation, other sterilization methods have been employed in the decontamination of medical cannabis such as beta irradiation (e-beam) and cold plasma [30].

Gamma irradiation is a form of electromagnetic energy that kills microorganisms throughout a product and its packaging. This technology, which offers deep penetration at low dose rates, was chosen as the most suitable for treating the multiple lots of National Institute on Drug Abuse (NIDA) and Drug Supply Program (DSP) plant material, as each lot varies somewhat in density and quantity. This treatment has little temperature effect on the product [31,32]. A given dose of gamma radiation takes time to deliver and, depending on the thickness and volume of the product, it may take minutes to hours [33]. The dose of gamma irradiation delivered to a packaged product unit is expressed in Kilo gray (KGY), the standard unit of measurement of ionizing radiation [28]. The irradiation process has been used commercially for more than forty years. To be effective, gamma irradiation needs time, contact, and temperature. The effectiveness of gamma irradiation is also dependent on the type of microorganism present [32]. Irradiation helps to reduce post-harvest losses through suppressing sprouting and contamination and enabling the eradication or control of insect pests, reductions in food-borne diseases, and the extension of shelf lives [34,35].

Gamma irradiation is used in the cannabis industry to develop biomass and products that are safe to consumers. With cannabis containing various types of bioactive phytochemicals including cannabinoids and terpenes (plus others) [36], it is important to ascertain the effect of gamma irradiation on these constituents.

According to the United States Pharmacopeia (USP), methods and specifications for the absence of *Salmonella* spp. and *Escherichia coli* are included in general chapter <62>, *Microbiological Examination of Nonsterile Products*. Meanwhile, the USP general chapter <61> includes the methods for enumerating total aerobic bacterial count and the total yeasts and molds count [37]. The Cannabis Expert Panel recommended specifications for the microbial quality control of cannabis plant material. The total aerobic bacterial count should be NMT 10^5^ CFU/g, and the total combined mold and yeast count should be NMT 10^4^ CFU/g, in addition to the absence of *Salmonella* spp. and *Escherichia coli* [38].

In this work, we aimed to determine the effect of irradiation on the microbial content (bacterial and fungal) and on the chemical and physical profile of the cannabis biomass, particularly the cannabinoids content, terpenes, and moisture content. Moreover, this study explores the effects of gamma irradiation on cannabis biomass, as this specific aspect of gamma irradiation’s effect on the major cannabinoids and terpenes may not have been extensively studied before compared to the previously published studies. Furthermore, testing the impact of gamma irradiation on moisture content in cannabis biomass may give an idea of the storage conditions of cannabis products, which represents an important area for the cannabis industry.

## 2. Results and Discussion

More than 2000 kg of cannabis plant material in 150 barrels was subjected to gamma irradiation to lower its microbial burden since these plant materials can be used in human clinical trials that might include immunocompromised subjects. All dosimetry results were in the range of 15.0 to 20.8 KGY (target range 10 to 30 KGY).

### 2.1. Microbial Decontamination Test Results

The microbial loads of three samples of cannabis plant material before and after gamma irradiation are displayed in Table 1. The Total Yeast and Mold Count (TYMC) was reduced from 13,000, 10,050, and 4500 CFU/g to less than 100 CFU/g, and the bacterial load (Total Aerobic Microbial Count (TAMC)) was also significantly reduced to less than 100 CFU/g by the gamma irradiation treatment. Although neither *E. coli* nor *Salmonella* spp. were detected in any lot before or after the irradiation treatment, these tests were included in the study because these tests are common in cannabis quality testing protocols.

### 2.2. Cannabinoid Acid Content

The concentrations of eleven cannabinoids (neutral and acid) including Δ^9^-THC, Δ^9^-THCA, CBD, CBDA, CBG, CBGA, Δ^8^-THC, CBN, CBC, CBCA, and THCV as % *w*/*w* were determined using our previously reported HPLC method [39].

**Figure 1:**
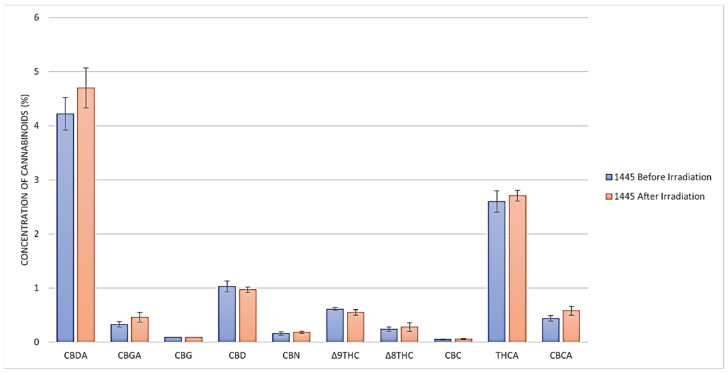
Cannabinoid content (% *w*/*w*) of a representative sample (# CS-445) of THC/CBD cannabis chemovar (before and after gamma irradiation using HPLC).

The cannabinoid content before and after the gamma irradiation treatment of a representative sample (#CS-445) is illustrated in Figure 1. No significant change (*p* > 0.05) in any of the cannabinoid concentrations was evident in any of the materials before and after irradiation. Δ^8^-THC was not detected before and after the irradiation of the THC/CBD cannabis chemovar.

### 2.3. Total Cannabinoid Content

**Figure 2:**
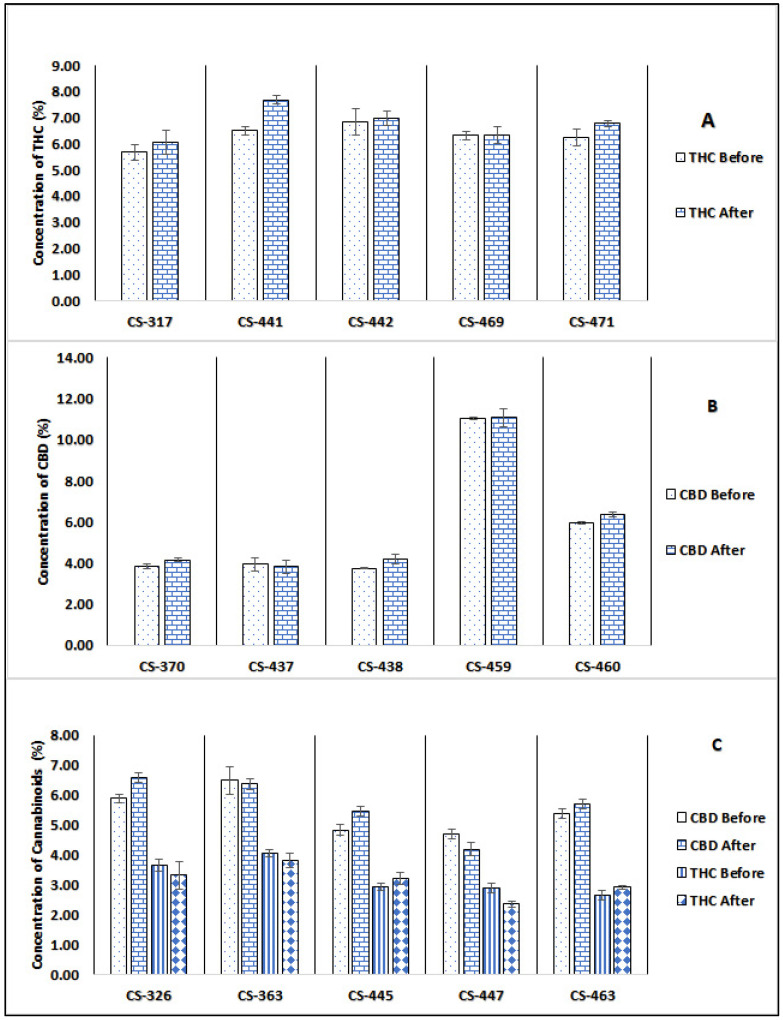
Cannabinoid content (% *w*/*w*) of (**A**)THC in high THC chemovar, (**B**) CBD in high CBD chemovar, and (**C**) THC and CBD in THC/CBD chemovar before and after gamma irradiation using GC/FID.

The results in Figure 2 and Table 2 show the THC and CBD concentrations for various cannabis batches of all three chemovars (high THC, high CBD, and THC/CBD chemovars), before and after the irradiation treatment. The total cannabinoid content was determined using our previously published method [40].

As demonstrated in Table 3, a paired *t*-test was employed to assess the variations in total cannabinoid concentrations within the samples before and after irradiation. The results indicate that there were no statistically significant changes, as all calculated *p*-values exceeded the 0.05 significance threshold (Table 3).

### 2.4. Terpene Content

**Figure 3:**
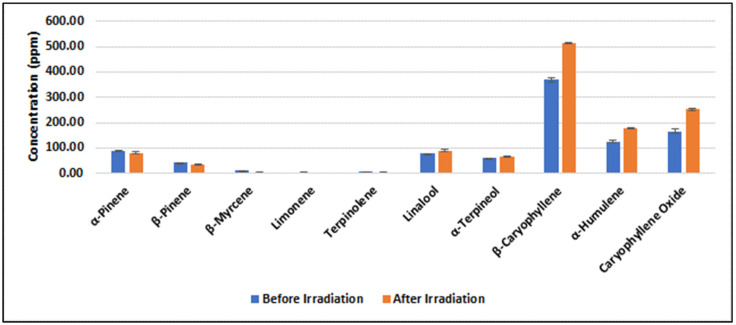
Terpene content (ppm) in a representative sample of high THC chemovar (sample #CS317) before and after gamma irradiation.

The quantitative analysis of terpenes was performed by GC/MS [41]. For a representative sample (#CS173, Figure 3), a paired *t*-test was utilized to evaluate the changes in terpene concentrations before and after irradiation. The analysis shows a *p*-value of 0.13, indicating that no statistically significant alteration in terpene concentration was observed within the sample, confirming the results of a previous study [29]. All samples were analyzed in triplicate.

### 2.5. Moisture Content

Among measurement standards for foodstuffs and agricultural products, Loss on Drying continues to be the most widely used method for moisture content [38]. The technical simplicity of the technique probably accounts for its popularity [38]. The results of moisture content are illustrated in Table 2.

The moisture content was calculated from the following equation:

$$Moisture\left(\%\right)=\frac{Mb-Ma}{Mb}\times 100$$

where *Mb* and *Ma* are the mass of the sample before and after drying, respectively. The difference denotes the moisture content of the sample. From the results, no significant change (*p* > 0.05) in the moisture content of any of the samples was observed due to the irradiation treatment (Table 3).

## 3. Materials and Methods

### 3.1. Cannabis Plant Material

Cannabis plants were grown in the field at the University of Mississippi, United States of America. Female cuttings of screened and selected high-yielding chemovars (high THC, THC/CBD, and high CBD) were grown indoors in four-inch biodegradable jiffy pots. Well-rooted cuttings were then transferred to an outdoor field for further growth. At maturity, plants were harvested, dried (for 24 h at 40 °C in a ventilated oven), processed, and stored in FDA-approved barrels. These barrels were stored at 25 °C for future use. Samples were taken from each barrel and ground in a stainless-steel coffee grinder and used for subsequent analyses.

### 3.2. Gamma Irradiation Treatment Procedures

Sterigenics is a contract sterilization company that operates a gamma irradiation facility located in West Memphis, Arkansas, 145 km from the National Center of Natural Products Research (NCNPR), at the University of Mississippi. The facility normally operates continuously, receiving commercial products in large shipments trucked to the facility for treatment. The Drug Enforcement Agency (DEA) Schedule-I control status of most of the DSP materials required special handling and security measures for the treatment, including authorizations by DEA, the National Institute on Drug Abuse (NIDA), and the Arkansas State Board of Pharmacy. The materials were transported in a cargo truck with security escorts.

The Sterigenics facility uses a hanging tote irradiator system with eighteen Cobalt-60 source stations. Each tote can accommodate products in packages of up to 30′ × 24′ × 40′ dimensions and 23 kg in weight. To prepare the packaged materials to fit the totes, the polyethylene bags containing the plant material were transferred to appropriate-size cardboard boxes before transportation to the facility. At the facility, a dosimeter was attached to each box which was then loaded onto a tote. The totes were carried by a conveyor system through a radiation shield into the irradiation chamber where they passed by the source stations following a serpentine route to expose all sides of the boxes to the gamma rays. The target dose for each box was specified to be in the range of 10 to 30 KGY based on recommendations by Sterigenics specialists. When the totes carrying the boxes exited the irradiation chamber after several hours of treatment, each dosimeter was read to verify that each box had received the specified dose.

#### 3.2.1. NIDA DSP Materials Currently Treated

As of August 2020, all the DSP plant material supplies stocked at NCNPR have undergone gamma irradiation treatment. A total of 2016 kg of plant material in 150 different barrel lots was treated on three different occasions.

#### 3.2.2. Microbial Contamination

Microbial testing was performed in duplicate following USP <61> and <62> procedures for Total Yeast and Mold Count (TYMC), Total Aerobic Microbial Count (TAMC), *E. coli*, and *Salmonella* spp. [38].

#### 3.2.3. Treatment Study

In order to measure the effect of gamma irradiation treatment on the chemical and physical characteristics of cannabis plant material, thirty-three barrel lots of the three cannabis chemovars were analyzed at NCNPR before and after the treatment for cannabinoids, terpenes, and moisture content. In order to verify the effectiveness of the treatment in reducing microbial contaminants, three barrel lots were tested for microbes before and after the treatment.

#### 3.2.4. Dose Verification

A scintillation counter was used to measure the exposure of each dosimeter to verify that each box had received the specified dose.

### 3.3. Cannabinoid and Terpene Standards

Cannabidiol (CBD), Cannabidiolic acid (CBDA), *trans*-Δ^9^-tetrahydrocannabinol (Δ^9^-THC), *trans*-Δ^9^-tetrahydrocannabinolic acid A (THCAA), Cannabigerol (CBG), Cannabigerolic acid (CBGA), *trans*-Δ^8^-tetrahydrocannabinol (Δ^8^-THC), cannabichromene (CBC), cannabinol (CBN), and *trans*-Δ^9^-tetrahydrocannabivarin (THCV) were isolated from different cannabis varieties at the University of Mississippi. Cannabichromic acid (CBCA) was purchased from Cayman^®^ (Ann Arbor, MI, USA). All cannabinoid standards were checked for their purity by GC/MS and HPLC (purity ≥ 99%). Terpene standards—*α*-pinene, *β*-pinene, *β*-myrcene, d-limonene, terpinolene, linalool, terpineol, *β*-caryophyllene, *α*-humulene, and caryophyllene oxide—were purchased from Sigma-Aldrich^®^ (Burlington, MA, USA). The purity of all standards was checked by GC-MS and found to be ≥ 90%, except terpinolene, which was ≥85%. *n*-tridecane (purity ≥ 99%) was used as the internal standard and purchased from Sigma-Aldrich^®^.

**Figure 4:**
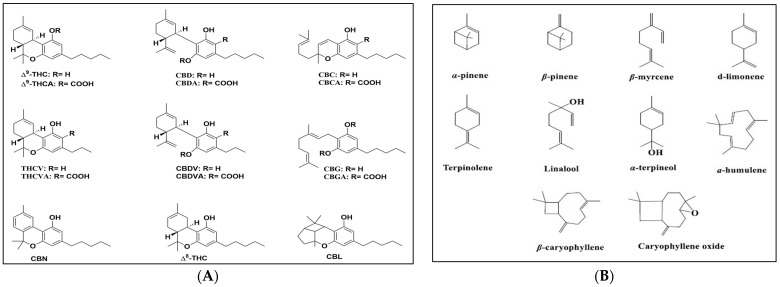
(**A**) Chemical structures of the tested cannabinoids and (**B**) terpenes.

The chemical structures of the tested cannabinoids and terpenes are shown in Figure 4.

### 3.4. Solvents and Reagents

The solvents including MeOH, ACN, and EtOAc were of HPLC grade and purchased from Fisher Scientific^®^ (Waltham, MA, USA). Chloroform was of analytical grade and purchased from Sigma-Aldrich^®^.

### 3.5. Determination of Total Cannabinoids Using GC-FID

The method used by NCNPR for the routine analysis of cannabinoids uses a gas chromatograph with a flame ionization detector (GC/FID) to quantitate seven different cannabinoids: Δ^9^-tetrahydrocannabinol (Δ^9^-THC), Δ^8^-tetrahydrocannabinol (Δ^8^-THC), cannabidiol (CBD), cannabinol (CBN), tetrahydrocannabivarin (THCV), cannabichromene (CBC), and cannabigerol (CBG) [40]. All 33 barrel lots were tested by this validated method which directly determines the “total cannabinoid” profile which reflects the cannabinoid amounts available to a person through smoking. All samples were analyzed in triplicate.

GC analyses were performed using a Varian CP-3380 gas chromatograph equipped with Varian CP-8400 automatic liquid samplers, capillary injectors, and dual-flame ionization detectors. The analysis was carried out using a DB-1MS column (15 m × 0.25 mm I.D., 0.25 µm film thickness; Agilent, Santa Clara, CA, USA). Data were acquired using a Dell Optiplex GX1 computer and Varian Star workstation software (version 6.1). Helium was used as the carrier and detector makeup gas at a flow rate of 1 mL/min with an upstream split ratio of 50:1. The injector temperature and the detector temperature were 240 °C and 270 °C, respectively. The temperature program was 170 °C (hold 1 min) to 250 °C at 10 °C/min (hold 3 min); run time, 12 min; injection volume, 1 µL. The instrument was calibrated daily to ensure a Δ^9^-THC/internal standard response factor ratio of one.

#### Calculation of Concentrations

The concentration of a specific cannabinoid was calculated as follows:

$$Cannabinoid\%=\frac{Area\;of\;the\;analyte}{Area\;of\;IS}\times \frac{Amount\;of\;IS}{Amount\;of\;sample}\times 100$$

In order to remove seeds and stems, the samples were manicured in a 14-mesh metal sieve. Triplicate samples (100 mg) were extracted with internal standard solution (IS) (3 mL, 4-androstene-3,17-dione in chloroform: methanol (100 mL, 1:9, *v*/*v*), 1 mg/mL) at room temperature for 1 h. The extracts were filtered through a cotton plug and then transferred to GC vials, ready for analysis. An aliquot of 1 μL was injected into the GC/FID.

### 3.6. Quantitative Analysis of Cannabinoid Acids Using HPLC-PDA

Cannabis samples were analyzed for both the free and acid forms of cannabinoids using a high-performance liquid chromatograph equipped with a photodiode array detector (HPLC/PDA), and eleven different cannabinoids were quantitated according to our validated method [39]. All samples were analyzed in triplicate. The chromatographic conditions are shown in Table 4.

Manicured plant samples (50 mg each) were weighed into glass scintillation vials and extracted each with 10 mL of I.S. solution (100 µg/mL) by sonication for 20 min at 30 °C. The samples were filtered through 45 µm nylon syringe filters. An aliquot of 10 μL of each sample was injected into the HPLC-PDA. All analyses were performed in triplicate.

### 3.7. GC/MS Analysis of Terpenes

The terpene profile was determined using the previously validated GC-MS method according to the following conditions [41]. The used chromatographic and mass spectrometric conditions are shown in Table 5.

#### Preparation of Standard and Sample Solutions for the Analysis of Major Terpenes

A stock standard solution of each terpene (*α*-pinene, *β*-pinene, myrcene, d-limonene, terpinolene, linalool, *α*-terpineol, *β*-caryophyllene, *α*-humulene, and caryophyllene oxide) was prepared in ethyl acetate. The standard terpenes were mixed, and the concentration of each terpene was adjusted to be 1.0 mg/mL, from which, serial dilutions were made to prepare the individual points of the calibration curves. Five calibration points ranging from 0.75 to 100 µg/mL were prepared from the previously mentioned stock standard solutions and IS (100 µg/mL).

Samples from three chemovars of *C. sativa* (high THC chemovar, THC/CBD chemovar, and high CBD chemovar) were dried for 24 h at 40 °C in a ventilated oven and then ground in a stainless-steel coffee grinder. Triplicates (1.0 g each) of the powdered samples were weighed in a 15 mL centrifuge tube, and each was extracted with 10 mL of the extraction solution (100 µg/mL of *n*-tridecane as the IS in ethyl acetate) by sonication for 15 min. The mixture was centrifuged for 5 min at 1252× *g*, and the supernatants (without filtration) were used for the GC/MS analysis.

### 3.8. Determination of Moisture Content

The moisture content of biomass is critical to the quality of stored cannabis plant material as excessive moisture promotes the growth of mold. Moisture content was measured for all 33-barrel lots in triplicate using a validated Loss on Drying (LoD) method [38].

### 3.9. Statistical Analysis

In this study, a paired *t*-test was conducted to compare the mean concentrations of cannabinoids and terpenes before and after gamma irradiation. The null hypothesis (H0) stated that there is no significant difference in the concentrations, while the alternative hypothesis (H1) posited the presence of a significant difference. A paired *t*-test was chosen due to its suitability for comparing paired data. The test was executed using the ‘paired *t*-test’ function in JASP software (version 0.18.1.0) with a significance level of α = 0.05.

## 4. Conclusions

The results suggest that the gamma irradiation treatment of bulk cannabis plant material does not significantly affect their chemical or physical properties but does effectively reduce microbial contamination to levels considered safe for human use. Although gamma irradiation treatment is practical for the decontamination of large quantities of materials, other technologies such as X-ray irradiation may be considered for smaller quantities of cannabis. Upon irradiation, cannabinoids, terpenes, and moisture content were not changed by the irradiation treatment.

## Figures and Tables

**Table 1:** Microbial test results, before and after irradiation treatment for three representative samples expressed as CFU/g.

	TYMC (CFU/g)		TAMC (CFU/g)		*E. coli* and *Salmonella* spp.	
Sample #	Before Irradiation	After Irradiation	Before Irradiation	After Irradiation	Before Irradiation	After Irradiation
**CS-464**	13,000	<10 *	1000	No growth	Absent/g	Absent/g
**CS-481**	10,050	<100 *	4100	<100	Absent/g	Absent/g
**CS-483**	4500	<100 *	3950	<100	Absent/g	Absent/g

CFU/g = colony-forming unit per gram. * Significant (*p* < 0.05). TYMC = Total Yeast and Mold Count. TAMC = Total Aerobic Microbial Count. # = number.

**Table 2:** Cannabinoid and moisture content (%*w*/*w*) of different cannabis chemovars after and before gamma irradiation (analyzed by GC/FID).

Sample #	Variety	CBD *		Δ^9^-THC *		CBN		THCV		CBC		CBG		Moisture Content	
		B	A	B	A	B	A	B	A	B	A	B	A	B	A
**CS-326**	**THC/CBD Chemovar**	5.97 ± 0.13	6.42 ± 0.16	3.64 ± 0.20	3.85 ± 0.45	0.29	0.33	0.04	0.04	0.28	0.31	0.36	0.40	7.73	8.03
**CS-363**		6.57 ± 0.45	6.49 ± 0.19	3.95 ± 0.11	3.85 ± 0.25	0.26	0.28	0.03	0.03	0.31	0.31	0.28	0.28	7.93	7.90
**CS-445**		4.65 ± 0.16	5.23 ± 0.16	2.82 ± 0.12	3.07 ± 0.20	0.24	0.23	0.03	0.03	0.25	0.21	0.29	0.25	8.05	8.22
**CS-447**		4.54 ± 0.16	3.96 ± 0.23	2.67 ± 0.18	2.27 ± 0.10	0.20	0.19	ND	ND	0.21	0.18	0.21	0.18	8.28	8.37
**CS-463**		5.32 ± 0.17	5.73 ± 0.16	2.73 ± 0.17	2.85 ± 0.06	0.37	0.43	ND	ND	0.23	0.25	0.22	0.24	8.1	8.23
**CS-464**		4.61 ± 0.27	5.03 ± 0.01	2.38 ± 0.10	2.48 ± 0.27	0.34	0.43	ND	ND	0.18	0.26	0.31	0.28	8.01	7.65
**CS-465**		4.75 ± 0.17	4.13 ± 0.08	1.97 ± 0.12	1.60 ± 0.10	0.40	0.35	ND	ND	0.20	0.18	0.21	0.18	8.14	8.47
**CS-466**		3.58 ± 0.15	4.12 ± 0.09	1.73 ± 0.15	1.94 ± 0.14	0.22	0.27	ND	ND	0.17	0.14	0.27	0.25	8.45	8.55
**CS-467**		3.43 ± 0.17	3.77 ± 0.08	1.25 ± 0.18	1.39 ± 0.11	0.25	0.28	ND	ND	0.16	0.13	0.11	0.11	8.18	8.10
**CS-370**	**High CBD Chemovar**	3.79 ± 0.12	4.06 ± 0.10	0.10 ± 0.08	0.11 ± 0.07	ND	ND	ND	ND	0.12	0.13	0.07	0.08	7.82	8.11
**CS-437**		3.86 ± 0.32	3.75 ± 0.30	0.18 ± 0.12	0.16 ± 0.09	0.02	0.01	ND	ND	0.14	0.16	0.14	0.16	8.66	8.35
**CS-438**		3.70 ± 0.04	4.00 ± 0.22	0.12 ± 0.10	0.12 ± 0.03	0.07	0.08	ND	ND	0.14	0.14	0.07	0.08	8.94	8.27
**CS-459**		10.96 ± 0.06	11.14 ± 0.45	0.39 ± 0.14	0.36 ± 0.03	0.06	0.05	0.03	0.03	0.36	0.36	0.32	0.29	8.04	8.12
**CS-460**		5.89 ± 0.05	6.22 ± 0.12	0.20 ± 0.15	0.18 ± 0.08	ND	ND	ND	ND	0.21	0.25	0.15	0.16	7.77	7.90
**CS-461**		5.74 ± 0.12	5.71 ± 0.05	0.16 ± 0.11	0.15 ± 0.04	ND	ND	ND	ND	0.20	0.20	0.12	0.12	7.75	8.05
**CS-468**		3.00 ± 0.12	3.17 ± 0.02	0.86 ± 0.16	0.87 ± 0.07	0.20	0.21	ND	ND	0.11	0.12	0.11	0.11	8.61	9.34
**CS-481**		4.70 ± 0.11	5.49 ± 0.07	0.35 ± 0.17	0.37 ± 0.08	0.03	0.03	0.01	0.01	0.24	0.27	0.12	0.14	8.00	8.16
**CS-483**		5.26 ± 0.13	5.44 ± 0.04	0.10 ± 0.08	0.10 ± 0.02	0.14	0.16	0.01	0.01	0.31	0.30	0.25	0.24	6.41	6.40
**CS-506**		4.50 ± 0.08	4.37 ± 0.02	0.20 ± 0.11	0.20 ± 0.01	0.03	0.04	0.01	0.01	0.30	0.24	0.09	0.08	5.64	6.28
**CS-317**	**High THC Chemovar**	0.09 ± 0.01	ND	5.61 ± 0.30	6.08 ± 0.44	0.16	0.23	0.04	0.04	0.10	0.10	0.08	0.10	10.47	10.18
**CS-441**		0.15 ± 0.04	0.16 ± 0.08	6.49 ± 0.16	7.52 ± 0.16	0.52	0.54	0.04	0.05	0.31	0.33	0.16	0.18	8.74	8.26
**CS-442**		0.06 ± 0.01	0.06 ± 0.01	6.58 ± 0.50	7.02 ± 0.28	0.53	0.64	0.07	0.07	0.28	0.32	0.13	0.19	7.28	7.65
**CS-469**		ND	ND	6.54 ± 0.18	6.60 ± 0.31	0.66	0.60	0.09	0.07	0.20	0.19	0.21	0.23	8.43	8.65
**CS-471**		ND	ND	6.12 ± 0.34	6.62 ± 0.12	0.54	0.57	0.05	0.04	0.19	0.16	0.18	0.17	7.44	8.14
**CS-472**		ND	ND	7.39 ± 0.10	5.97 ± 0.02	0.70	0.66	0.05	0.04	0.26	0.23	0.28	0.23	8.51	8.04
**CS-473**		ND	ND	5.49 ± 0.04	5.45 ± 0.12	0.52	0.58	0.04	0.04	0.22	0.19	0.16	0.16	7.97	7.95
**CS-474**		ND	ND	4.97 ± 0.05	5.24 ± 0.02	0.49	0.58	0.03	0.03	0.18	0.19	0.14	0.15	7.92	8.13
**CS-475**		ND	ND	5.78 ± 0.15	5.87 ± 0.09	0.50	0.55	0.04	0.04	0.21	0.20	0.17	0.18	8.44	8.01
**CS-476**		ND	ND	3.40 ± 0.18	3.91 ± 0.05	0.46	0.44	ND	ND	0.16	0.18	0.11	0.13	7.32	7.30
**CS-477**		ND	ND	6.01 ± 0.13	5.22 ± 0.10	0.57	0.55	0.05	0.04	0.22	0.19	0.18	0.16	7.9	7.52
**CS-478**		ND	ND	2.42 ± 0.07	2.63 ± 0.15	0.27	0.31	ND	ND	0.15	0.15	0.06	0.07	7.56	7.87
**CS-479**		ND	ND	2.22 ± 0.06	2.50 ± 0.09	0.28	0.35	ND	ND	0.16	0.19	0.06	0.06	7.85	7.85
**CS-480**		0.05 ± 0.01	0.05 ± 0.04	1.44 ± 0.14	1.32 ± 0.03	0.44	0.39	0.04	0.04	0.13	0.15	0.04	0.04	7.58	7.81

# = number. **B** = before irradiation, **A** = after irradiation, **ND** = not detected, **Δ^8^-THC** was not detected in any sample. * Concentration % *w*/*w* ± standard error.

**Table 3:** Paired sample *t*-test of total cannabinoids in cannabis samples analyzed by GC/FID.

Before Irradiation	After Irradiation	t	df	*p*-Value
CS-326B	CS-326A	−2.354	6	0.057
CS-363B	CS-363A	1.571	6	0.167
CS-445B	CS-445A	−1.506	6	0.183
CS-447B	CS-447A	1.475	5	0.200
CS-463B	CS-463A	−2.116	5	0.088
CS-464B	CS-464A	−0.487	5	0.647
CS-465B	CS-465A	0.946	5	0.388
CS-466B	CS-466A	−1.621	5	0.166
CS-467B	CS-467A	−1.069	5	0.334
CS-370B	CS-370A	−1.782	4	0.149
CS-437B	CS-437A	1.286	4	0.268
CS-438B	CS-438A	0.444	5	0.676
CS-459B	CS-459A	−0.933	6	0.387
CS-460B	CS-460A	−1.551	4	0.196
CS-461B	CS-461A	−0.835	4	0.450
CS-468B	CS-468A	−1.313	5	0.246
CS-481B	CS-481A	−1.332	6	0.231
CS-483B	CS-483A	−0.925	6	0.391
CS-506B	CS-506A	−0.658	6	0.535
CS-317B	CS-317A	−0.451	5	0.671
CS-441B	CS-441A	−0.525	6	0.619
CS-442B	CS-442A	−2.116	6	0.079
CS-469B	CS-469A	−0.865	5	0.427
CS-471B	CS-471A	−1.508	5	0.192
CS-472B	CS-472A	1.475	5	0.200
CS-473B	CS-473A	0.344	5	0.745
CS-474B	CS-474A	−2.076	5	0.092
CS-475B	CS-475A	0.621	5	0.562
CS-476B	CS-476A	−0.996	4	0.376
CS-477B	CS-477A	1.598	5	0.171
CS-478B	CS-478A	−1.838	4	0.140
CS-479B	CS-479A	−1.445	4	0.222
CS-480B	CS-480A	−0.281	6	0.788

**Table 4:** HPLC/PDA conditions for the analysis of cannabinoid acids.

Parameter	Description
HPLC instrument	Waters Alliance 2695e HPLC system with a binary HPLC pump and a Waters 2996 PDA detector.
Column and guard column	Luna C18(2) column (150 × 4.60 mm, 3 µm; Phenomenex, Torrance, CA) equipped with a C18 guard column cartridge (Phenomenex).
Mobile phase	of 0.1% (*v*/*v*) formic acid in water (mobile phase A) and 0.1% (*v*/*v*) formic acid in acetonitrile (mobile phase B) according to a gradient elution started at 70% B from 0 to 6 min; then 77% B in 6 min; kept 77% B for 10 min; afterwards, the system was returned to the initial conditions with a total run time of 22.2 min.
Flow rate	1.2 mL/min.
Injection volume	10 µL.
PDA wavelength	220 nm.
Software	Empower 3 software.

**Table 5:** GC/FID parameters for the analysis of cannabis terpenes.

Parameter	Description
Instrument/ Software	Agilent 7890A series (Agilent) GC. Software (NIST) (Version 2.0f; Standard Reference Data Program of the National Institute of Standards and Technology, as distributed by Agilent Technologies).
Column	DB-5MS capillary column (30 m × 0.25 mm I.D., 0.25 µm film thickness; Agilent).
Carrier gas	Helium; flow rate of 1 mL/min.
Inlet temperature/ Split mode	250 °C/split ratio 15:1.
Injection volume	2 µL.
Temperature program	The temperature program initiated at 50 °C (held for 2 min), then increased to 85 °C at a rate of 2 °C/min, followed by a ramp to 165 °C at 3 °C/min. Post-run, the temperature was held at 280 °C for 10 min.
Mass conditions	Full scan mode; from 40 to 450 atomic mass units (amu). The ionization energy = 70 eV. Ion source temperature = 230 °C. Quadrupole temperature = 150 °C. Solvent delay was set to 4 min. Transfer line temperature was 280 °C. Total run time was 56.16 min.

## Data Availability

Data are contained within the article.
